# Protein tyrosine phosphatase nonreceptor type 22 (PTPN22) gene single nucleotide polymorphisms and its interaction with T2DM on pulmonary tuberculosis in Chinese Uygur population

**DOI:** 10.18632/oncotarget.19274

**Published:** 2017-07-15

**Authors:** Xian-Hua Wang, Ai-Guo Ma, Xiu-Xia Han, Lei Chen, Hui Liang, Aishan-Litifu, Abudumijit-Ablez, Feng Xue

**Affiliations:** ^1^ The School of Public Health, Medical College of Qingdao University, Qingdao, P.R China; ^2^ Department of Respiratory Medicine, Xinjiang Uygur Autonomous Region Chest Hospital, The Xinjiang Uygur Autonomous Region, Urumqi, P.R China; ^3^ Tuberculosis Department of The Xinjiang Uygur Autonomous Region Center for Disease Control and Prevention, The Xinjiang Uygur Autonomous Region, Urumqi, P. R China

**Keywords:** pulmonary tuberculosis, *PTPN22*, single nucleotide polymorphisms, interaction, T2DM

## Abstract

**Aims:**

To investigate the association of several single nucleotide polymorphisms (SNPs) within Protein tyrosine phosphatase nonreceptor type 22 (*PTPN22*) gene and additional gene- gene and gene- type 2 diabetes mellitus (T2DM) interaction with pulmonary tuberculosis (PTB) risk in Chinese Uygur population.

**Methods:**

A total of 722 participants (186 males, 536 females) were selected, including 360 PTB patients and 362 control participants. Generalized multifactor dimensionality reduction (GMDR) was used to screen the best interaction combination among SNPs and T2DM. Logistic regression was performed to investigate association between 3 SNPs within *PTPN22* gene, additional gene- gene and gene- T2DM interaction on PTB risk.

**Results:**

Logistic regression analysis showed that PTB risk was significantly lower in carriers with rs2476601- CT genotype than those with CC genotype (CT versus CC), adjusted OR (95%CI) =0.42 (0.17-0.83), and higher in carriers with the rs33996649- GA genotype than those with GG genotype (GA versus GG), adjusted OR (95%CI) = 5.66 (2.24-9.47). We found a significant two-locus model (p=0.0010) involving rs33996649 and T2DM. Overall, the cross-validation consistency of this two- locus model was 10/ 10, and the testing accuracy was 60.11%. We also conducted stratified analysis for rs33996649 and T2DM using logistic regression. We found that T2DM patients with rs33996649 - GA genotype have the highest PTB risk, compared to non- T2DM patients with rs33996649- GG genotype, OR (95%CI) = 4.52 (2.71 -6.43), after covariates adjustment.

**Conclusions:**

We found that the T allele of rs2476601 and the A allele of rs33996649within *PTPN22* gene, interaction between rs2476601 and T2DM were all associated with increased PTB risk.

## INTRODUCTION

Pulmonary tuberculosis (PTB), caused by Mycobacterium tuberculosis, is one of the deadliest infectious diseases worldwide [1]. According to the World Health Organization (WHO), in 2011, there were an estimated 8.7 million new cases of TB and 1.4million died [2]. China ranks the second among the 22 high-burden countries, with different incidence and prevalence in different provinces [3, 4]. The prevalence rate of PTB was significant different among several ethnic minorities [5], therefore, the differences in susceptibility to TB may be related to a genetic predisposition. Several studies have reported PTB related genetic factors previously [6–8].

Protein tyrosine phosphatases were involved in maintaining the T cells in the resting stage and are also responsible for bringing back the activated T cells to the resting phenotype in the absence and presence of antigen, respectively [9, 10]. The *PTPN22* gene was located on chromosome 1p13.3-p13.1 and encoded intracellular lymphoid tyrosine phosphatase (LYP) [11], which was expressed only in cells of hematopoietic origin, dephosphorylates kinases. Several studies have reported a significant association between single nucleotide polymorphisms (SNPs) and susceptibility to several autoimmune diseases, such as type 1 diabetes, rheumatoid arthritis and systemic lupus erythematosus (SLE) [12–14].

PTB risk was influenced by not only genetic factors, but also some environment factors. In all modifiable risk factors, type 2 diabetes mellitus (T2DM) was an important environmental risk factor for PTB, and PTB incident was also influenced by gene- environment interactions, but to date, less study focused on the impact of *PTPN22* gene SNPs, interaction between *PTPN22* gene SNPs and T2DM on PTB susceptibility. So the aim of this study was to investigate the impact of several SNPs within *PTPN22* gene, and their additional gene- gene interaction, gene-T2DM interaction on PTB risk, based on a Chinese population.

## RESULTS

A total of 722 participants (186 males, 536 females) were selected, including 360 PTB patients and 362 control participants. The mean age of all participants was 43.9 ± 11.4 years. Table 1 shows the general characteristics in cases and controls. The means of age and BMI, distribution of males were not significantly different between cases and controls. The prevalence of T2DM was higher in cases than controls.

**Table 1:** General characteristics for all study participants in PTB cases and controls

Variables	PTB cases (n=360)	Controls (n=362)	*P-values*
Age (years)	43.5±11.6	44.2±12.1	0.428
Males N (%)	91 (25.3)	95 (26.2)	0.767
BMI (kg/m^2^)	22.7±9.2	23.1±9.6	0.568
Type 2 diabetes, N (%)	148 (41.1)	107 (29.6)	<0.001
Clinical feature, N (%)			
Haemoptysis	124 (34.4)		
Expectoration	320 (88.9)		
Fever	340 (94.4)		
Cough	356 (98.9)		
Abnormality shown by chest X-ray examination, N (%)	360 (100)		

BMI, body mass index; N, number

No significant difference in genotype frequencies from the Hardy–Weinberg equilibrium test was noted for any tested SNPs in the controls. The frequency for the CT of rs2476601 genotype was significantly lower in PTB cases than controls (1.9% *vs*4.7%), and contrarily, the frequency for the GA genotype of rs33996649was significantly higher in PTB cases than controls (7.5% *vs*2.8%). Logistic regression analysis showed that PTB risk was significantly lower in carriers with rs2476601- CT genotype than those with CC genotype (CT versus CC), adjusted OR (95%CI) =0.42 (0.17-0.83), and higher in carriers with the rs33996649- GA genotype than those with GG genotype (GA versus GG), adjusted OR (95%CI) = 5.66 (2.24-9.47). However, we did not find any direct association of the rs2488457 with PTB risk after covariates adjustment. (Table 2)

**Table 2:** Genotype and allele frequencies of 3 SNPs between case and control group

SNP	Genotypes and alleles	Frequencies N (%)		OR (95%CI)*	*P-* values ^b^	*P-* values for HWE test in controls
		Controls (n=362)	Cases (n=360)			
*R620W* (C1858T) (rs2476601)						
	Co-dominant					
	CC	345 (95.3)	353 (98.1)	1.00 (ref)		0.647
	CT	17 (4.7)	7 (1.9)	0.42 (0.17-0.83)	<0.001	
	TT	0 (0)	0 (0)	-		
	Dominant					
	CC	352(97.2)	333 (92.5)	1.00 (ref)		
	CT+TT	10 (2.8)	27 (7.5)	5.66 (2.24-9.47)	<0.001	
	Allele, T (%)	0 (0)	0 (0)			
*R263Q* (G788A) (rs33996649)						
	Co-dominant					
	GG	352(97.2)	333 (92.5)	1.00 (ref)		0.790
	GA	10 (2.8)	27 (7.5)	5.66 (2.24-9.47)	<0.001	
	AA	0 (0)	0 (0)	-		
	Allele, A (%)	148 (20.4)	223 (31.0)			
rs2488457 (-1123G>C)						
	Co-dominant					
	GG	207 (57.2)	185 (51.4)	1.00 (ref)		0.249
	GC	128 (35.4)	139 (38.6)	1.20 (0.79-1.74)	0.429	
	CC	27 (7.5)	36 (10.0)	1.41 (0.82-2.06)	0.536	
	Dominant					
	GG	207 (57.2)	185 (51.4)	1.00 (ref)		
	GC+CC	155 (42.8)	175 (48.6)	1.22 (0.80-1.84)	0.408	
	Allele, C (%)	182 (25.1)	211 (29.3)			

*Adjusted for gender, age, T2DM and BMI. ^b^*p*<0.0083 (Bonferroni correction threshold)

We also investigate the synergistic effect among 3 SNPs and T2DM using GMDR model. Table 3 summarized the results obtained from GMDR analysis. We found a significant two-locus model (p=0.0010) involving rs33996649 and T2DM, indicating a potential interaction between rs33996649 and T2DM on PTB risk. Overall, the cross-validation consistency of this two- locus model was 10/ 10, and the testing accuracy was 60.11%. We also conducted stratified analysis for rs33996649 and T2DM using logistic regression. We found that T2DM patients with rs33996649 - GA genotype have the highest PTB risk, compared to non- T2DM patients with rs33996649- GG genotype, OR (95%CI) = 4.52 (2.71 -6.43), after covariates adjustment (Table 4).

**Table 3:** GMDR analysis on the best gene–gene and gene- T2DM interaction models

Locus no.	Best combination	Cross-validation consistency	Testing accuracy	*p-values*
Gene- gene interactions^*^				
2	rs2476601 rs33996649	8/10	0.5399	0.0547
3	rs2476601 rs33996649 rs2488457	7/10	0.5399	0.1719
Gene- T2DM interactions ^**^				
2	rs33996649 T2DM	10/10	0.6011	0.0010
3	rs33996649 rs2476601 T2DM	8/10	0.5399	0.1719
4	rs33996649 rs2476601 rs2488457 T2DM	7/10	0.4958	0.3770

*Adjusted for gender, age, T2DM and BMI.

**Adjusted for gender, age and BMI.

**Table 4:** Stratified analysis for gene- T2DM interaction by using logistic regression

rs2476601	T2DM	OR (95% CI)^*^	*P-values*
GG	No	1.00	-
GA	No	1.31 (1.02 -1.70)	0.036
GG	Yes	1.42 (1.16-1.87)	<0.001
GA	Yes	4.52 (2.71 -6.43)	<0.001

*Adjusted for gender, age, drinking and BMI

## DISCUSSION

In this study, we found that both the T allele of rs2476601 and the A allele of rs33996649were significantly associated with PTB risk in a sample of the Chinese Uygur population. PTB risk was significantly lower in carriers with rs2476601- CT genotype than those with CC genotype, but higher in carriers with the rs33996649- GA genotype than those with GG genotype. However, we did not find any direct association of the rs2488457 with PTB risk after covariates adjustment. Several similar studies on association between *PTPN22* SNP and PTB risk have been performed. In contrast to our findings, Lamsyah et al [15] observed statistically significant differences in the *PTPN22* C1858T genotypic and allelic frequencies between PTB and controls in a Moroccan population. The frequency of the minor allele (T allele) was found to be 0.41% in PTB and 3.2% in normal subjects. In another study, Gomez et al [16] found statistically significant differences in the *PTPN22* C1858T genotypic and allelic frequencies between PTB and controls in a Columbian population. The frequencies of the T allele were found to be 1.3% in PTB and 4.3% in normal subjects. The distribution of T allele frequencies in our study was in accordance with these findings. Kouhpayeh et al [17] indicated that frequencies of genotypes CC, CT and TT of the *PTPN22* C1858T polymorphism were 98.3%, 1.7% and 0% in the pulmonary tuberculosis patients, and 96.1%, 3.9% and 0% in the control group, respectively. The frequency of the minor (T) allele was 0.8% in pulmonary tuberculosis patients and 2.0% in controls, but they found no significant differences were observed in genotype or allele frequencies of *PTPN22* C1858T in the comparison between pulmonary tuberculosis patients and healthy subjects in an Iranian population sample. Narasimha et al [8] conducted a study for Indian population and indicated that the frequencies of genotypes CC, CT, and TT of rs2476601 were 100%, 0%, and 0%, respectively, in PTB cases; and 99.2%, 0.8% and 0%, respectively, in healthy control individuals, however, these values were not significantly different between the patients and controls.

PTB susceptibility was influenced by both genetic and environment factors, and previously some environmental factors associated with PTB were reported, such as smoking, diabetes, anti-TNFa drugs and poor glycemic control [18–20], and in these risk factors, T2DM has been suggested to play a crucial role in increasing the risk of PTB risk. In current study, the rate of T2MD was higher in PTB cases than controls, so we also investigated gene- gene interaction and gene- environment interaction between SNPs and T2DM. We found a significant interaction between rs33996649 and T2DM on PTB risk (p=0.0010). Overall, the cross-validation consistency of this two- locus model was 10/ 10, and the testing accuracy was 60.11%. T2DM patients with rs33996649 - GA genotype have the highest PTB risk, compared to non- T2DM patients with rs33996649- GG genotype. The mechanisms by which DM increases TB risk were not well understood. Several early studies reported reduced pro-inflammatory cytokines in mouse models of diabetes mellitus after infection with *Mycobacterium tuberculosis*, and others found that cytokine responses were consistent with a Th-2 phenotype [21, 22].

There several limitations in our study. Firstly, just three SNPs were included in this study, more SNPs should been included in the future. Secondly, some others environmental risk factors should be included in the gene- environment interaction detection, such as smoking, BMI and so on. Thirdly, sex differences on this association should be investigated in the future studies with larger sample size.

In conclusion, we found that the T allele of rs2476601 and the A allele of rs33996649within *PTPN22* gene were associated with increased PTB risk. We also found a significant interaction between rs2476601 and T2DM on PTB risk, T2DM patients with rs33996649 - GA genotype have the highest PTB risk, compared to non- T2DM patients with rs33996649- GG genotype.

## MATERIALS AND METHODS

### Subjects

The study consisted of 360 PTB patients (91 males, 269 females; mean age: 43.5±11.6 years) and 362 healthy subjects (95 males, 267 females; mean age: 44.2±12.1 years). All PTB patients were evaluated by microbiological diagnosis (microscopic and culture examination), medical history, physical examination and chest X-ray. All patients were positive at sputum smear and/or culture assays. Each subject (patients and controls) enrolled in this study was negative for HIV-1/2 infection (assayed by Axsym Assays; Abbott Laboratories, Chicago, IL). The controls were randomly selected from a population, who received physical examination in our hospital and 1:1 matched to cases on the basis of age (±3 years) and sex. Individuals recruited to the health control group had no history of TB. Both the PTB cases and controls were unrelated Chinese Uygur population. Questionnaire investigation was conducted for all participants, and data on demographic information, clinical and biochemical index for all participants were obtained. Body weight and height were measured. Blood samples were collected from each participant in the morning after at least 8 hours of fasting. Informed consent was obtained from all participants.

### Genomic DNA extraction and genotyping

**Figure 1:**
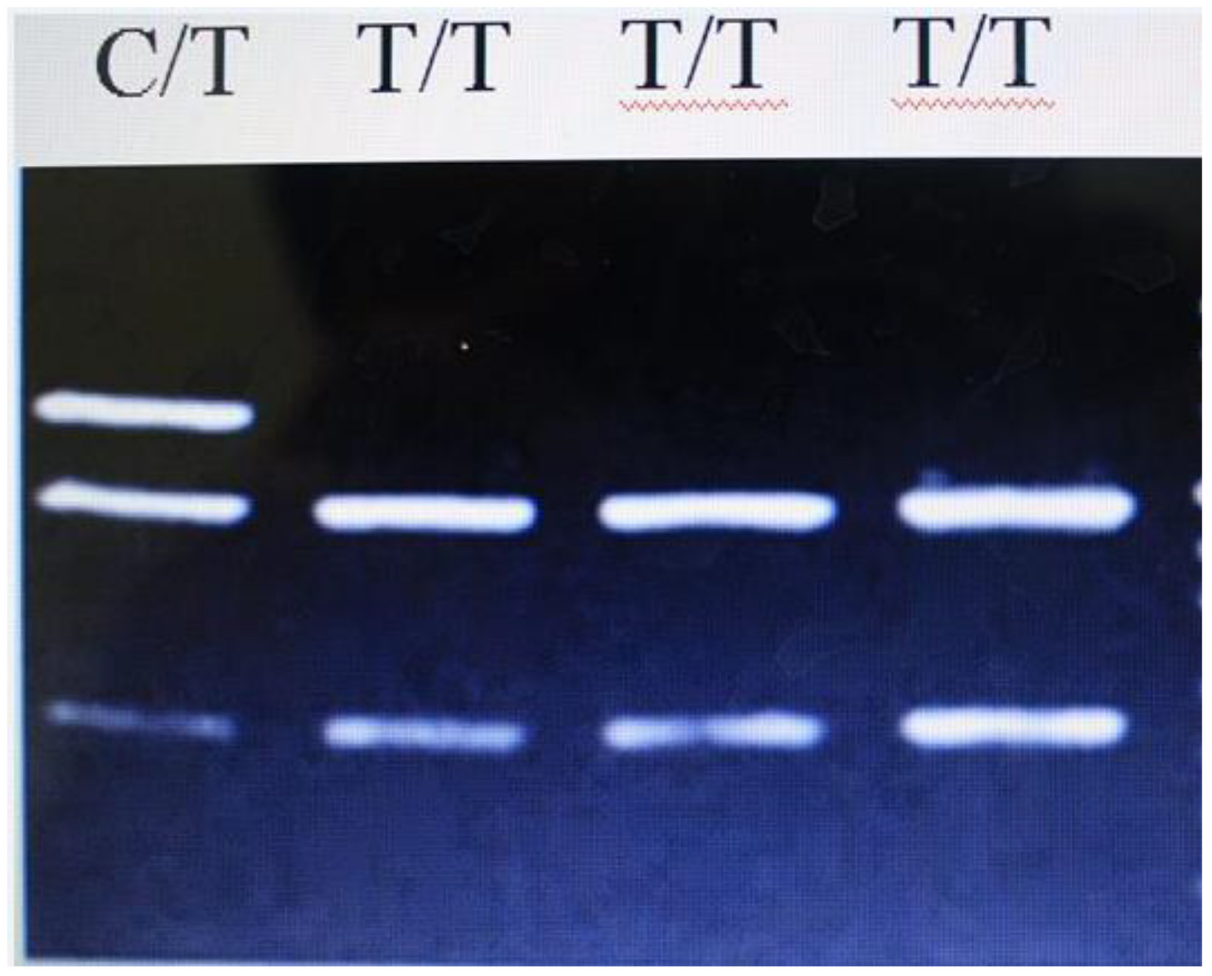
Results of PCR product enzyme digestion for rs2476601

The SNPs were selected based on the NCBI database (http://www.ncbi.nlm.nih.gov/projects/SNP). Taking into account the limited human resources and financial resources, just 3 SNPs within *PTPN22* gene were selected for genotyping, including: rs2476601, rs33996649 and rs2488457. Genomic DNA from participants was extracted from EDTA-treated whole blood, using the DNA Blood Mini Kit (Qiagen, Hilden, Germany) according to the manufacturer’s instructions. The three SNPs were tested using PCR–RFLP analysis, followed by agarose gel electrophoresis [23]. Primers for the all SNPs and the corresponding endonuclease are listed in Table 5. PCR amplification was carried out in a 1*PCR buffer with 2 mM MgCl_2_, 200 μM dNTP, 0.4 μM of each primer, 15 ng of genomic DNA and 0.25 IU of Taq polymerase within a final volume of 10 μl. The following cycling conditions were used: initial denaturation step of 2 min at 95°C; 35 cycles of 30 s at 95°C, 25 s at 59°C, 53°C, 59°C (for rs2476601, rs33996649, rs2488457 as annealing temperature, respectively); 30 s at 72°C and final extension step of 3 min at 72°C, results of PCR product enzyme digestion for rs2476601 was shown in Figure 1. Genotyping results were confirmed by randomly assaying 10% of the original specimens for replication to exclude genotyping errors. There were no discrepancies between genotypes determined in duplicate.

**Table 5:** Description and primer sequence for 3 SNPs used for PCR analysis

SNP ID	Chromosome	Functional consequence	Major/ minor alleles	Restriction enzyme	Primer sequences
*R620W* (C1858T) (rs2476601)	1:113834946	Intron variant, missense	C/ T	RsaI	F: 5’-ACTGATAATGTTGCTTCAACGG-3’ R: 5’-TCACCAGCTTCCTCAACCAC-3’
*R263Q* (G788A) (rs33996649)	1:113852067	Intron variant, missense	G/ A	MspI	F: 5’-GATGGAGCAAGACTCAGACAC-3’ R: 5’-CCCCATGTTAGAAGAGCAGAT-3’
rs2488457	1:113872746	Intron variant, upstream variant 2KB	G/ C	SacI	F: 5′-CCATTGAGAGGTTATGCGAGCT-3′ R: 5′-CGCCACCTTGCTGACAACAT-3′

### Statistical analysis

Categorical variables were presented as absolute values and percentages, and continuous variables were expressed as mean ± standard deviation (SD). Student’s t test was used to compare continuous variables, while Chi-square test was used to compare categorical variables between cases and controls. Hardy-Weinberg equilibrium (HWE) examination was used by SNPstats (http://bioinfo.iconcologia.net/SNPstats). Logistic regression was performed to investigate the impact of 3 SNPs within *PTPN22* gene on PTB risk, and additional stratified analysis for gene- T2DM interaction on PTB risk. Two sided test with *P* < 0.05 was considered statistically significant.

Generalized multifactor dimensionality reduction (GMDR) [24] was used to screen the best interaction combination among SNPs and T2DM., the cross-validation consistency, testing balanced accuracy and the sign test, to assess each selected interaction were calculated. The cross-validation consistency score was a measure of the degree of consistency with which the selected interaction was identified as the best model among all possibilities considered. The testing balanced accuracy was a measure of the degree to which the interaction accurately predicts case–control status with scores between 0.50 (indicating that the model predicts no better than chance) and 1.00 (indicating perfect prediction). Finally, a sign test or a permutation test (providing empirical p-values) for prediction accuracy can be used to measure the significance of an identified model.
